# Combined electroacupuncture and auricular acupuncture for postoperative pain after abdominal surgery for gynecological diseases: study protocol for a randomized controlled trial

**DOI:** 10.1186/s13063-017-2359-8

**Published:** 2018-01-04

**Authors:** Wing Lok Lam, Wing Fai Yeung, Mei Kuen Wong, Chi Wai Cheung, Karen Kar Loen Chan, Hextan Yuen Sheung Ngan, Carlos King Ho Wong, Hai Yong Chen, Lixing Lao

**Affiliations:** 1 School of Chinese Medicine, The University of Hong Kong, 10 Sassoon Road, Pokfulam, Hong Kong, Special Administrative Region of China; 2 School of Nursing, the Hong Kong Polytechnic University, Kowloon, Hong Kong, Special Administrative Region of China; 3 Department of Anesthesiology, The University of Hong Kong, 4/F, K Block, Queen Mary Hospital, 102 Pokfulam Road, Pokfulam, Hong Kong, Special Administrative Region of China; 4 Department of Obstetrics and Gynecology, The University of Hong Kong, 6/F, Professorial Block, Queen Mary Hospital, 102 Pokfulam Road, Pokfulam, Hong Kong, Special Administrative Region of China; 5 Department of Family Medicine and Primary Care, The University of Hong Kong, 3/F, Ap Lei Chau Clinic, 161 Main Street, Ap Lei Chau, Pokfulam, Hong Kong, Special Administrative Region of China

**Keywords:** RCT, Health economic analysis, Placebo, Analgesia, Morphine consumption, AUC, Acupuncture, Operation

## Abstract

**Background:**

Postoperative pain is a major complaint following abdominal surgery for gynecological diseases. Effective postoperative pain control after abdominal surgery is particularly important for patient recovery, mobility, and satisfaction. Acupuncture has been commonly used for pain management. However, its efficacy in postoperative pain control is inconclusive and health economic evaluation is limited.

**Methods:**

A randomized, sham-controlled, patient- and- assessor-blind trial is designed to evaluate the efficacy and safety of acupuncture in managing postoperative pain following abdominal surgery of gynecological diseases. Patients who are eligible for laparotomy with a midline incision for gynecological neoplasia, including ovarian mass, uterine mass, and cervical lesions, will be recruited. Seventy-two participants will be randomly allocated to an acupuncture or non-invasive sham control in a 1:1 ratio. Treatment will be done within 2 h before operation, upon arrival to the ward and once daily for 5 days. The Pain Numerical Rating Scale (NRS) on the first 5 days during hospitalization will be the primary outcome and will be analyzed using the area-under-the-curve (AUC) method. The secondary outcome measures include frequency of rescue analgesic use during hospital stay, cumulative morphine consumption; quality of recovery as measured by time to recovery variables and the Quality of Recovery-9 (QoR-9); quality of life as measured by the Short Form-6 dimension (SF-6D) and EuroQol-5 Dimension-5 Level (EQ-5D-5 L). The incremental cost-effectiveness ratio of acupuncture vs sham acupuncture will be calculated.

**Discussion:**

This study protocol provides an example of integrative medicine practice in a hospital setting for the management of postoperative pain using acupuncture treatment. The acupuncture treatment protocol, if proven to be effective, can be implemented in routine settings to play a role in postoperative pain management for patients who have undergone abdominal surgery for gynecological diseases.

**Trial registration:**

ClinicalTrials.gov, ID: NCT02851186. Registered on 19 July 2016.

**Electronic supplementary material:**

The online version of this article (doi:10.1186/s13063-017-2359-8) contains supplementary material, which is available to authorized users.

## Background

Postoperative pain is a major complaint following abdominal surgery for gynecological diseases. Effective postoperative pain control after abdominal surgery is particularly important for patient recovery, mobility, and satisfaction. Pain at the intra-abdominal incision site may interfere with deep breathing which is necessary for early ambulation. If the acute pain is inadequately treated, it may become chronic; surgery contributes to chronic pain in 23% of patients in outpatient pain clinics [1]. Pharmacological agents, such as epidural anesthesia, intraspinal anesthesia, intrapleural anesthesia, or patient-controlled analgesia (PCA), are effective in relieving postoperative pain in most patients. However, analgesic drugs, especially opioid analgesics, are associated with complications including drowsiness, pruritus, nausea and vomiting and respiratory depression. Thus, a safe and effective treatment for postoperative pain is needed.

Acupuncture is defined as needling at acupoints on the human body surface, including the trunk, limbs, scalp, and auricle. Acupuncture has been commonly used for pain management in the Asian population. Its analgesic effect has been well documented. Emerging evidence shows that acupuncture’s analgesic effects involves a variety of bioactive chemicals through peripheral, spinal and supraspinal mechanisms [2]. Acupuncture can induce endogenous opioids which desensitize peripheral nociceptors and reduce proinflammatory cytokines in peripheral tissues and the spinal cord. Acupuncture also suppresses *N*-methyl-d-aspartate receptors (NMDAR) by inducing endogenous opioids in the intrarostral anterior cingulate cortex of the brain, which contributes to the inhibition of the affective dimension of pain [2].

Several studies have shown that acupuncture can relieve pain after gynecological surgeries [3–5], abdominal surgeries [6–8], and other types of surgeries including oral surgery [9], thoracotomy [10], cardiac surgery [11], and laparoscopic cholecystectomy [12–14]. There have been six systematic reviews of acupuncture or complementary therapies for postoperative pain. Four of the six reviews [15–18] suggested that acupuncture is potentially useful in the management of postoperative pain. In the review of acupuncture on postoperative pain after back surgery conducted by Cho et al. [15], a pooled meta-analysis of five high-quality trials showed that acupuncture reduced Visual Analog Scale (VAS) pain scores (standard mean difference (SMD) = 0.67 (95% confidence interval (CI) 1.04 to 0.31)), but not opioid consumption (SMD = − 0.23 (95% CI − 0.58 to 0.13)), when compared to sham control at 24 h following back surgery. In a meta-analysis by Sun et al. [16], the acupuncture group showed a significantly lower cumulative opioid analgesic consumption at 8, 24, and 72 h (weighted mean difference (WMD) = – 3.14 mg (95% CI − 5.15 to − 1.14), − 8.33 mg (95% CI − 11.06 to − 5.61), and − 9.14 mg (95% CI − 16.07 to − 2.22), respectively) and pain level at 8 and 72 h (WMD = – 14.57 mm (95% CI − 23.02 to − 6.13) and − 9.75 mm (95% CI − 13.82 to − 5.68), respectively) in comparison to sham control after a wide range of operations, including abdominal surgery. Asher et al. [17] performed a review on auriculotherapy, suggesting that auriculotherapy is an effective intervention in relieving perioperative pain as suggested by the reduced analgesic use (SMD 0.54 (95% CI 0.30 to 0.77)). Another systematic review on auricular acupuncture by Usichenko et al. [18] found that the evidence of auricular acupuncture in reducing postoperative pain is promising (eight out of nine included studies supported the use of auricular acupuncture when compared to control, but no meta-analysis was done due to the heterogeneity of the studies) but not compelling due to poor methodological quality of studies. Although the use of acupuncture in postoperative pain control was not supported either review [19, 20], these reviews focused on pain management after ambulatory knee surgery [19] and orthopedic surgery [20] which may be quite different from abdominal surgery.

Electroacupuncture (EA) at PC6, ST36 bilaterally, and along the skin incision subcutaneously is better than sham EA in relieving postoperative abdominal pain as measured by postoperative morphine consumption in 6–12 h after operation in the study by Sim [3]. Zeng et al. (2014), found that the analgesic effect as measured by total pain remission rates calculated from VAS at 2, 4, and 6 h after treatment in the acupuncture group were better than those in the orally administered morphine sulphate group (63.3% vs 50%; 73.3% vs 66.7%, and 100% vs 76.7%, respectively) [8]. Auricular acupuncture (AA), a variant of acupuncture, is also widely used for the management of pain by Chinese medicine practitioners. A previous systematic review concluded that the evidence on AA reducing postoperative pain is promising [18]. Therefore, acupuncture has a potential role in the management of postoperative pain.

Although previous studies have suggested that acupuncture and AA may be useful in relieving postoperative pain, the results are not compelling due to high risk of bias associated with improper randomization, inadequate control, and lack of blinding and allocation concealment. High-quality evidence is needed to confirm the previous findings and support the use of acupuncture for postoperative pain in the routine clinical setting.

We, therefore, propose a randomized, double-blind, controlled trial using a rigorous methodology, adequate allocation concealment, and validated outcome measures to examine the efficacy of acupuncture as an adjunct treatment in relieving postoperative pain after abdominal surgery for gynecological diseases. To maximize the efficacy of acupuncture treatment, a combined approach of EA and AA, which is commonly used in clinical practice, will be used.

We hypothesized that acupuncture, as an adjunct treatment, is effective and safe to reduce postoperative pain during the first 5 days following abdominal surgery for gynecological diseases as compared to a sham control. The overall objective of this study is to examine the safety and efficacy of a combined acupuncture treatment of electroacupuncture (EA) and intradermal auricular acupuncture (AA) as an adjunct treatment in reducing postoperative pain in patients after abdominal surgery for gynecological diseases, compared with sham EA in combination with sham AA. Specifically, to test the hypothesis, the objectives of the study are:

1. To determine the efficacy of acupuncture as an adjunct treatment in reducing postoperative pain as measured by the pain Numerical Rating Scale (NRS, the primary outcome) and pain medication consumption during the first 5 days after operation to be calculated as the area under the curve (AUC) to compare the between-group difference
2. To determine whether the acupuncture treatment can shorten the time to recovery as measured by time to recovery variables including time to recovery of postoperative bowel functions and food intake; time to ambulation and time to discharge
3. To evaluate the safety of acupuncture treatment in postoperative pain in a hospital ward by comparing the number of subjects with adverse events, number of patients withdrawn and reasons of withdrawal in treatment group with the sham control group
4. To assess whether acupuncture is cost-effective in improving the postoperative quality of life of patients using the Quality of Recovery-9 (QoR-9), Short Form-6 dimension (SF-6D), and EQ-5D-5 L when compared with sham acupuncture

## Methods

### Study design

The proposed study will be a randomized, parallel-group, sham-controlled, subject- and assessor-blind trial (number of sample, *n* =72). The trial will be commenced after ethical approval has been obtained from the Institutional Review Board. All study-related procedures will be performed only after subjects have given their written informed consent. The clinical trial is designed and reported following the Consolidated Standards of Reporting Trials (CONSORT) guidelines, the Standard Protocol Items: Recommendations for Interventional Trials (SPIRIT) Checklist (Additional file 1), and Standards for Reporting Interventions in Controlled Trials of Acupuncture (STRICTA) recommendations.

### Participants

#### Sample size estimation

Results from an earlier study of EA as an adjunct treatment in postoperative pain were used for sample size consideration [10], and the expected mean difference and standard deviations of AUC in 5 days for the treatment group and control group were obtained by simulation using R [21].

The mean difference of AUC between treatment group and sham group in the previous study [10] is 3.4 with standard deviations of 2.9 for the treatment group and 3.6 for the sham group (effect size = 1.04). A lower limit of the 70% confidence interval of the effect size which is 0.75 is applied [22]. A sample size of 30 per group can provide 80% power to reject the null hypothesis with a significance level of 0.05 using a two-sided, two-sample, unequal-variance *t* test. With an expected dropout rate of 15%, the final sample size is 72 (36 per group). AUC is used to calculate the primary outcome measure because it provides information regarding the pain relief across the whole period, which is more meaningful than comparing the pain score at a particular time point. AUC also has an advantage in that it avoids multiple comparisons and thus will not inflate type I error. This statistical analysis approach has been widely used and reported by previous studies, including those published in leading pain journals [23, 24].

#### Subject recruitment

**Fig. 1:**
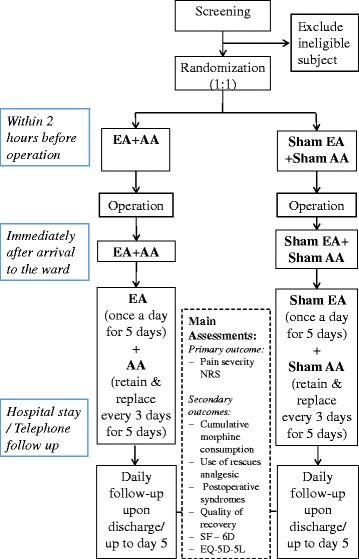
Study flowchart. Abbreviations: *EA* electroacupuncture, *AA* auricular acupuncture

**Fig. 2:**
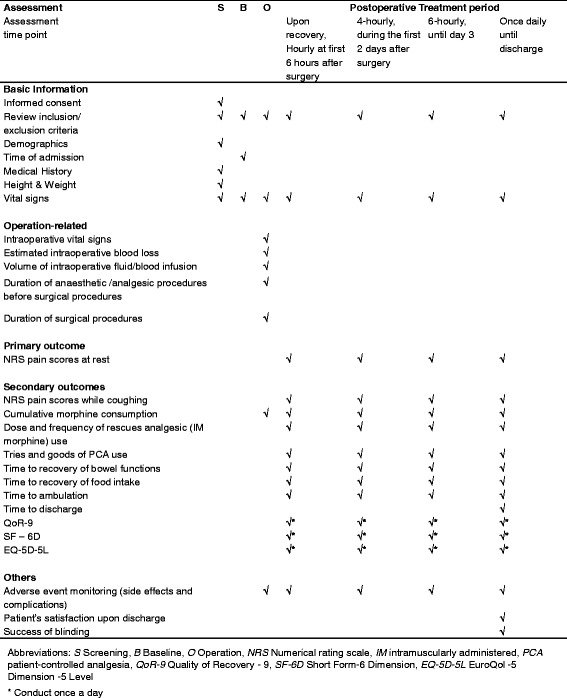
SPIRIT - Figure: Study assessment schedule

This randomized, sham-controlled, double-blind (subject- and assessor-blind) trial will be conducted in the Department of Obstetrics and Gynecology, at Queen Mary Hospital. Potentially eligible subjects who have scheduled for abdominal surgery for gynecological diseases will be invited to participate. Patients will be referred from a surgeon or gynecologist, and then a research assistant will approach the subjects at the pre-admission clinic or in the general ward pre operation. Patients will be screened and consented for the study. Following the consent, eligible participants will be block randomized into two groups: (I) acupuncture (*n* = 36) and (II) sham acupuncture (*n* = 36). All participants will undergo a standard operative procedure and receive postoperative analgesics according to usual practice. Treatment will be given within 2 h prior to the surgery, immediately after arrival on the ward, and daily following surgery for 5 days. Assessments will be conducted upon recovery, hourly at 6 h after surgery, then 4-hourly until day 2 and then 6-hourly until discharge. (See Fig. 1 for the study flowchart, and Fig. 2 for the assessment schedule).

#### Inclusion criteria

1. American Society of Anesthesiologists (ASA) physical status I to III
2. Eligible for laparotomy with midline incision for gynecological neoplasm including ovarian mass, uterine mass, and cervical lesions
3. Age 18 years or above
4. Ability to understand the nature of the study and willing to give informed consent
5. Capable of providing responses during outcome measurement

#### Exclusion criteria

1. Having chronic pain and using any medication in addition to the anesthetic and analgesia prescribed prior to surgery that would be expected to affect the dosage of postoperative analgesia
2. History of or current alcohol or drug abuse
3. Impaired renal function, defined as preoperative serum creatinine level over 120 μmol/L
4. Impaired hepatic function, defined as preoperative serum albumin level below 30 g/L
5. Impaired or retarded mental state and not able to sign the consent
6. Not self-ambulatory before operation
7. Difficulties in using patient-controlled analgesia
8. Body Mass Index > 35 kg/m^2^
9. Infection observed at the acupoint sites
10. History of acupuncture experience in the previous 12 months
11. History or clinical evidence of valvular heart defects, heart failure, atrial fibrillation, or bleeding disorders or are fitted with a cardiac pacemaker or taking anticoagulant drugs

### Randomization

Block randomization with a random block size ranging from 4 to 8 will be used in this study. Participants are randomly assigned to one of the two groups in 1:1 ratio after completion of baseline assessment, according to a randomized block list generated by an independent research assistant using R and the code will be kept in opaque sequentially numbered envelopes.

### Allocation concealment

The treatment allocation codes will be enclosed by an independent research assistant in sequentially numbered opaque envelopes and will not be revealed until the participants have completed all baseline assessments and immediately before the first acupuncture treatment. To minimize breaks in coding, multiple allocation codes will be assigned to both groups. The principal investigator (PI) who designed the trial, the co-investigators (Co-Is) who perform the on-site screening, and research personnel who perform the outcome assessments will be blinded to the treatment assignment.

### Blinding

Participants will be told that they will be randomly assigned to either acupuncture treatment or acupuncture-like sham treatment. The treatment procedure of acupuncture treatment and sham acupuncture treatment will be identical except that no skin penetration is involved in sham acupuncture (the details will be described in the “Study design – Sham acupuncture group” section). Therefore, participants will be unaware of the treatment that they received.

The principal investigator, other co-investigators, the nurse, the research assistant responsible for data collection and data entry and the statistician responsible for data analysis will be blinded to the participants’ group allocation.

Only the acupuncturist will know the participants’ group allocation. However, the acupuncturist will not know any other information about the patients such as the degree of the surgery, the amount of anesthetic used during the surgery, and analgesics consumed post surgery. To avoid accidental disclosure of group allocation by the acupuncturist, their interactions with the participants will be kept to a minimum.

### Quality assurance of acupuncture treatment

To ensure the quality of acupuncture treatment, pre-job training and examination will be provided by the principal investigator (Prof. L Lao) to the acupuncturists. The first treatment for the first five patients will be assessed by an expert in acupuncture (appointed by the principal investigator) who will confirm the location of the acupoints and the depth of needling as accurate with a checklist.

### Treatment protocol

The acupuncture treatment will be conducted within 2 h before surgery, after surgery at the ward and every 24 h after the surgery for 5 days (seven treatment sessions in total). A registered acupuncturist preferably with a Master’s degree in acupuncture and at least 3 years’ acupuncture experience will be responsible for delivering the acupuncture treatment.

### Acupuncture treatment group

#### Preoperative acupuncture treatment (first acupuncture session) and acupoint justification

Bowel preparation begins 1 day before the operation. Participants will fast from midnight before the operation. Within 2 h before the operation, participants will receive the first acupuncture treatment, which consists of auricular acupuncture (AA) using filiform needles and electroacupuncture (EA).

The acupuncture procedure will be performed following the Hospital Authority (HA) Guideline on Safety in Acupuncture for Chinese Medicine Practitioners [25]. AA will be performed in the supine position. Following skin preparation with 75% alcohol swabs, sterilized, disposable, filiform needles, each 15 mm long and 0.30 mm in diameter, will be inserted through a needle-guiding tube at the bilateral auricular acupoints *Shenmen* and a reflex point of the pain site corresponding to the abdominal region (four points in total). The needle-guiding tube will be removed. A piece of adhesive tape will then be immediately applied to the surface of the auricle. The reflex point will be detected with an acupoint detector, which works by detecting the low electrical resistance of the auricular acupoints. The auricular acupoint selection is based on previous studies [26, 27] and the traditional auriculotherapy theory of using corresponding anatomical body areas on the ears [28]. After that, a screening curtain will be placed on a rack above the participant’s chest to prevent the patient from watching the treatment procedure. Acupuncture points (see Table 1): bilateral LI4 (*Hegu*), ST36 (*Zusanli*), SP8 (*Diji*), SP10 (*Xuehai*), and LR3 (*Taichong*) will be used in all sessions. In addition, bilateral LR14 (*Qimen*), ST25 (*Tianshu*), EX-CA1 (*Zigong*), and unilateral CV4 (*Guanyuan*) and CV12 (*Zhongwan*) will also be used in the first session. All acupoints will be located with reference to the most recent acupuncture textbook of the International Standard Library of Chinese Medicine written by Zhang, Zhao, and Lao [29]. A 75% alcohol swab will be used to sterilize the points and skin around. Sterilized, disposable filiform needle, each 25–30 mm long and 0.30 mm in diameter, will be inserted at each point through a needle-guiding tube with reference to the suggested technique written in the textbook [29]. The needle-guiding tube will be removed after the insertion of needles. The needles will be manipulated to initiate a sensation of “*De qi*.” An electric stimulator (AWQ-104 L) will then be connected to eight pairs of needles: LI4-ST36, SP8-LR3, bilateral LR14, bilateral ST25, CV4-CV12, and bilateral EX-CA1. A low-frequency, 4-Hz continuous wave will be selected. Treatment will be carried on for 30 min. After the removal of needles, clean cotton wool will be used to prevent bleeding by compressing the points. The selection of acupoints is based on the principle of traditional acupuncture practice with a history of thousands of years [28, 29], previous clinical trials [3–5, 12, 13, 30, 31], and experts’ opinions (Prof. L Lao). A total number of 18 acupoints will be used in the first treatment and 14 acupoints will be used in the subsequent treatments. The acupoint selection and rationale are summarized in Table 1.

**Table 1:** Justification of acupoint in electroacupuncture for postoperative pain control based on Traditional Chinese Medicine (TCM)

	Location	TCM indication	Suggested technique
Core acupoints (*used in all treatment sessions*)			
LI4 (*Hegu*)	Dorsum of hand, at the level of the midpoint of the second metacarpal bone, between first and second metacarpal bones	Helps in abdominal pain.	Needle perpendicularly 0.5–1.0 cun
ST36 (*Zusanli*)	Antero-lateral leg, 1 middle-finger breadth next to the anterior crest of tibia, 3 cun under the depression lateral to the patellar ligament	Stomach’s lower *He* point, helps in regulating *Qi* and blood circulation.	Needle perpendicularly 0.5–1.5 cun
SP8 (*Diji*)	Medial leg, posterior to the tibia, 3 cun under the depression inferior and posterior to the tibia medial condyle	*Xi* (Cleft) point of the Spleen meridian, helps in abdominal pain	Needle perpendicularly 1.0–1.5 cun
SP10 (*Xuehai*)	Antero-medial thigh, 2 cun above the medial cranial pole of the patella.	Helps in tonifying and circulating blood	Needle perpendicularly 1.0–2.0 cun
LR3 (*Taichong*)	Dorsum of foot, within the depression between first and second metatarsal bones.	Helps in regulating liver *Qi* and blood	Needle obliquely upward 0.5–1.0 cun
Additional acupoints: *Before surgery (first treatment session): LR14, ST25, EX-CA1, CV4*, and *CV12 will be used. After surgery (second to seventh treatment sessions): 4 acupoints selected from below will be used, PC6 is the first choice*			
GB26 (*Daimai*)	Lateral abdomen, at the level of the umbilicus, below the free end of the 11th rib	Crossing point of the Gallbladder and the *Dai* meridians	Needle obliquely 0.5–1.0 cun
GB34 (*Yanglingquan*)	Lateral leg, at the depression inferior and anterior to the head of the fibula	*He* (Sea) point of the Gallbladder meridian and Influential Point for the tendons	Needle obliquely upward 0.5–0.8 cun
GB41 (*Zulinqi*)	Lateral-dorsal aspect of the foot, lateral side of the little toe’s extensor muscle tendon	*Shu* (Stream) point of the Gallbladder meridian and the meridian’s Confluence Point with the *Dai* meridians	Needle perpendicularly 0.5–0.8 cun
HT3 (*Shaohai*)	Elbow, at the middle of the medial end of the transverse antecubital crease and the medial epicondyle of the humerus	*He* (Sea) point of the Heart meridian, helps in regulating heart	Needle perpendicularly 0.5–1.0 cun
HT7 (*Shenmen*)	Palmar aspect of the wrist, ulnar end of the transverse crease	*Shu* (Stream) and *Yuan* (Source) points of the Heart meridian, helps in calming the *Shen* (spirit)	Needle perpendicularly 0.3–0.5 cun
KI4 (*Dazhong*)	Medial foot, at the back of and below the medial malleolus, in depression in front of the Achilles tendon	Helps in gynecological disorders	Needle perpendicularly 0.5–1.0 cun
KI6 (*Zhaohai*)	Medial foot, at the depression below the medial malleolus	Helps in gynecological disorders	Needle perpendicularly 0.5–0.8 cun
LI11 (*Quchi*)	Elbow, at the middle of *Chize* and the lateral epicondyle of the humerus	Helps in abdominal pain	Needle perpendicularly 1.0–2.5 cun
LR5 (*Ligou*)	Medial leg, 5 cun on top of the medial malleolus.	*Luo* (Connecting) point of the Liver meridian, helps in gynecological disorders	Needle transversely 0.5–0.8 cun
LR14 (*Qimen*)	Thorax, on the mammillary line, in the six intercostal space under the mammilla	Front Collecting Point (*Mu* point) of the Liver meridian, helps in regulating liver *Qi* and blood	Needle transversely 0.5–1.0 cun
LU7 (*Lieque*)	Antero-lateral forearm, in front of the styloid process of the radius, 1.5 cun away from the wrist crease	Confluence Point of the *Ren* meridian and the Lung meridian	Needle obliquely upward 0.2–0.3 cun
PC6 (*Neiguan*)	Palmar aspect of the forearm, between the tendons, 2 cun away from the transverse crease of the wrist	*Luo* (Connecting) point of the Pericardium meridian, helps in gastric pain and calming the *Shen*	Needle perpendicularly 0.5–1.5 cun
SP6 (*Sanyinjiao*)	Behind the medial tibia, 3 cun above the tip of the medial malleolus	Helps in gynecological disorders	Needle perpendicularly 0.5–1.0 cun
SP14 (*Fujie*)	Abdomen, 4 cun away from the anterior midline, 1.3 cun below *Daheng*	Helps in peri-umbilical pain	Needle perpendicularly 1.0–1.5 cun
SP15 (*Daheng*)	Abdomen, 4 cun away from the umbilicus	Helps in abdominal pain	Needle perpendicularly 1.0–1.5 cun
SP16 (*Fuai*)	Abdomen, 4 cun away from the anterior midline, 3 cun above *Daheng*	Helps in abdominal pain	Needle perpendicularly 1.0–1.5 cun
SP21 (*Dabao*)	Lateral chest, in 6th intercostal space, on mid-axillary line	Collateral meridian of Spleen, helps in total body pain	Needle obliquely backward 0.5–0.8 cun
ST23 (*Taiyi*)	Abdomen, 2 cun above *Tianshu*	Helps in reducing irritability	Needle perpendicularly 1.0–1.5 cun
ST25 (*Tianshu*)	Abdomen, 2 cun away from the navel	Front Collecting Point (*Mu* point) of the Large Intestine meridian, helps in circulating *Qi*	Needle perpendicularly 1.0–1.5 cun
ST27 (*Daju*)	Abdomen, 2 cun below *Tianshu*	Helps in lower abdominal distention and pain	Needle perpendicularly 1.0–1.5 cun
ST34 (*Liangqiu*)	Anterior thigh, 2 cun above the superior lateral corner of the patella	*Xi* (Cleft) point of the Stomach meridian, helps in acute stomachache	Needle perpendicularly 1.0–1.5 cun
CV4 (*Guanyuan*)	Abdomen, 3 cun under the navel	Front Collecting Point (*Mu* point) of the Large Intestine meridian, helps in invigorates original *Qi* (*Yuan Qi*)	Needle perpendicularly 0.5–1.0 cun
CV12 (*Zhongwan*)	Abdomen, 4 cun above the navel	Front Collecting Point (*Mu* point) of the Stomach meridian, Master Point of the *Fu* organs, helps in regulating *Qi*	Needle perpendicularly 0.5–1.0 cun
EX-CA1 (*Zigong*)	Abdomen, at the level of 4 cun under the navel and 3 cun away from the midline	Helps in gynecological disorders	Needle perpendicularly 0.8–1.2 cun

#### Postoperative acupuncture treatment (second to seventh acupuncture sessions) and acupoint justification

After the operation, the patient will be transferred to the ward after about 1 h. The subject will receive the second acupuncture treatment on the arrival to the ward. Afterwards, the subject will receive an acupuncture treatment every 24 h after the operation in the following 5 days (third to seventh acupuncture sessions). From the second acupuncture session onwards, a combined treatment approach of EA and AA using AA needles will be used.

The treatment procedure of EA, including needling procedure and electric-stimulation parameters, will be the same as the first acupuncture treatment described above, except that only four acupoints will be chosen from the additional acupoints list: GB26, GB34, GB41, HT3, HT7, KI4, KI6, LI11, LR5, LR14, LU7, PC6, SP6, SP14, SP15, SP16, SP21, ST23, ST25, ST27, ST34, CV4, CV12, and EX-CA1 with PC6 as the first choice. After completion of EA, AA will be performed as the first acupuncture treatment mentioned previously, but AA needles will be used. AA needles inserted will be retained and replaced every 3 days until discharge (in case of discharging before day 5) or day 5.

### Sham acupuncture group

To control for the non-specific effects of acupuncture, a non-invasive sham acupuncture method will be performed 1–2 cun away from the true acupoints, where no meridians cross, [32, 33] in the control group.

#### Procedure

To ensure standardization of the needling technique and how we convey to the subjects about “sham acupuncture,” all acupuncture will be performed by the same registered Chinese medicine practitioner. A backup practitioner will also be trained in case the primary practitioner is unavailable.

The acupuncture treatment procedure, including the acupoints selection principle, sterilization procedure, and treatment schedule (treatment time point, duration, and total number of treatments) will be the same as the treatment group except that non-invasive, retractable. sham needles, an inactivated electric stimulator, a non-invasive, sham-acupuncture procedure and dummy auricular intradermal needles will be used on points 1–2 cun away from the true acupoints where no meridians cross [32, 33]. Non-invasive, retractable, sham needles designed by Streitberger will be used [34] for sham EA. The sham needles are blunt needles that will not penetrate the skin during needle insertion. The handles of these sham needles will slide over the needle when it is compressed, giving the appearance of a needle penetrating the skin. The needles are held by surgical tape to imitate the acupuncture procedure. The needles will be connected to an electric stimulator with zero frequency and amplitude. For the sham AA in the first session, a validated sham-acupuncture procedure [35] will be adopted with small modification. A plastic needle-guiding tube will be taped to the points on the auricle to stimulate some discernible sensation. A needle with a piece of adhesive tape will then be immediately applied to the surface of the auricular acupoint [35]. For the sham AA from the second session onwards, dummy auricular intradermal needles with only circular handles will be used. The dummy auricular intradermal needles will be covered by adhesive tape.

### Anesthesia, intraoperative care, and postoperative management

#### Anesthesia

No participant will receive sedative premedication. On arrival in the operating theatre, a 16-gauge intravenous cannula will be inserted under local analgesia. Propofol 2 mg/kg, fentanyl 1.5 μg/kg, and atracurium 0.5 mg/kg will be used for general anesthesia induction. A bolus dose of 0.1 mg/kg morphine sulphate will be given intravenously prior to incision. Pulse, pulse oximetry, and non-invasive blood pressure measurement will be checked every 5 min throughout the operation.

#### Intraoperative care

Anesthesia will be maintained with a mixture of sevoflurane, air, and oxygen. The sevoflurane concentration will be titrated to optimal heart rate and blood pressure by the anesthesiologist (Dr. CW Cheung and his team members). At hours 3 and 4, morphine sulphate 0.1 mg/kg may be given in divided dose titrated to the patient’s response.

A thermal blanket and infusion fluid warmer will be used to maintain core body temperature at 35.5 to 37.5 °C. A urinary catheter will be inserted to monitor urine output. An intermittent pneumatic compression device will be applied to the lower limbs for deep vein thrombosis prophylaxis.

At the discretion of the anesthesiologist (Dr. CW Cheung and his team members), hypotension will be treated with intravenously administered ephedrine or phenylephrine. Hypertension or tachycardia will be treated with isoflurane up to 2% end-tidal concentration (approximately 1.5 MAC). If persistent, beta-blockers, such as labetalol, or arterial vasodilators, such as hydralazine, may be given. Sevoflurane will be switched off after the closure of the inner layer of the wounds. All participants will have local analgesia infiltrated to their wounds at the end of operation. Reversal will be achieved by intravenously administered neostigmine 50 μg/kg and atropine 20 μg/kg after the operation. The patient will then be transferred to the recovery room for monitoring then to the ward after about 1 h.

#### Postoperative management

All participants will be prescribed paracetamol 1 g every 6 h for 3 days and Celebrex 200 mg twice daily for 3 days. On arrival in the recovery room, boluses of 2 mg of intravenously administered morphine will be given every 5 min until the patient’s NRS pain score is less than 4. The patient will be offered patient-controlled analgesia (PCA). The PCA pump will be configured to give 1 mg of morphine at a time and the lockout duration will be set to 5 min. No basal infusion will be given and the maximum dose limit will be 0.1 mg/kg/h.

From postoperative day 0, rescue analgesic in the form of intramuscularly administered morphine 0.1 mg/kg will be prescribed every 4 h if necessary. During the administration of PCA morphine, the patient will be seen by an anesthesiologist, who is blinded to treatment allocation (Dr. CW Cheung and his team members), every day to assess the adequacy of analgesia.

PCA morphine will be administered for at least 2 days. On postoperative day 2, if the participant’s NRS pain score during cough is less than 4 on a clinically low morphine consumption, PCA will be stopped; if the NRS pain score is 4 or above, or her PCA consumption is still high, it will be continued. Assessment will be repeated daily. If a patient’s NRS pain score during a cough remains at 4 or above on day 5, she will be evaluated for complications and managed at the discretion of the anesthesiologist (Dr. CW Cheung and his team members).

### Measures

#### Outcome assessment

Subjects will be closely monitored during and after the operation. The primary outcome will be participants’ postoperative pain scores at rest measured by Numerical Rating Scale (NRS) for the first 5 days after operation. The secondary outcomes include postoperative NRS pain scores while coughing; dose and frequency of rescue analgesic use during hospital stay; time to recovery variables; QoR-9, SF-6D, and EQ-5D-5 L. If at any time point after the operation, a participant is unable to be managed according to the protocol for any reason, such as the development of complications, no further data will be collected. Telephone follow-up will be offered for participants discharged before day 5 (Fig. 2).

### Primary outcome measure

#### Pain intensity by Numerical Rating Scale (NRS) at rest

The NRS for pain is a 0–10-point scale with 0 indicating no pain and 10 indicating worst possible pain. NRS pain scores will be measured at rest upon recovery, hourly at 6 h after surgery, then 4-hourly until day 2, 6-hourly until day 3 and then once daily until discharge. The NRS is a commonly used scale for clinical pain research. NRS is chosen because it is easier for patients to grade their pain intensity with numbers and it has been shown to correlate well with the Visual Analog Scale and Brief Pain Inventory as well as other pain measurements [36–39].

### Secondary outcome measures

#### Pain intensity by Numerical Rating Scale (NRS) while coughing

The NRS for pain while coughing will be measured hourly at 6 h after surgery, then 4-hourly until day 2, then 6-hourly until day 3 and then once daily until discharge.

#### Cumulative morphine consumption

Postoperative PCA morphine consumption will be recorded in each participant’s medication log during the hospital stay.

#### Dose and frequency of rescue analgesic (intramuscularly administered morphine) use

The time to first postoperative use, dose, and frequency of rescue analgesic will be monitored and recorded in each subject’s medication log during the hospital stay.

#### Time to recovery variables

The time to recovery of postoperative bowel function and food intake; time to ambulation and time to discharge will be assessed and recorded by physicians during the hospital stay.

#### Quality of Recovery-9 (QoR-9)

The QoR-9 is a nine-item questionnaire, which has been extensively utilized and validated to assess the patients’ quality of recovery after anesthesia [40].

#### Short Form-6 dimension (SF-6D) and EuroQol -5 Dimension-5 Level (EQ-5D-5 L)

Quality of life will be assessed by using a Chinese-translated and validated Medical Outcomes SF-6D and EQ-5D-5 L. The SF-6D and EQ-5D-5 L are a preference-based measure of health (PBMH) for economic evaluation. The SF-6D is composed of six multi-level dimensions, namely, physical functioning, role participation, social functioning, bodily pain, mental health, and vitality, whereas the EQ-5D-5 L has five dimensions for health status description: mobility, self-care, usual activities, pain/discomfort, and anxiety/depression. The SF-6D and EQ-5D-5 L value set [41, 42] for the Chinese population will be used to derive utility scores.

### Side effects associated with opioid use

The occurrence of opioid side effects, including pruritus, sedation, dizziness, nausea, and vomiting and urinary retention, will be accessed.

### Safety assessment

#### Reasons for withdrawal

When a participant withdraws before completing the study, the reasons for withdrawal will be recorded.

#### Adverse events

Adverse events occurring during the study period will be recorded. Vital signs, including heart rate, respiratory rate and blood pressure, will be assessed. The researcher will interview participants using an adverse event record form daily during the hospital stay.

### Credibility assessment

The Credibility of Treatment Rating Scale will be used to assess the credibility of the acupuncture treatments. This four-item scale is specially designed to assess the credibility of acupuncture [43]. It assesses participants’ “confidence in the treatment to alleviate their complaint,” “confidence in recommending the treatment to their friends who have similar complaints,” “perceived logic of the treatment,” and “likelihood that the treatment would alleviate their other complaints.”

### Blinding success assessment

After the final treatment, the success of blinding will be tested by asking participants in the acupuncture treatment group and the sham acupuncture group the following question: “When you volunteered for the trial, you were informed that you had an equal chance of receiving traditional acupuncture or acupuncture-like simulation treatment. Which acupuncture do you think you received?” Three response options will be provided for patients to choose from: acupuncture treatment, acupuncture-like simulation treatment, and uncertain. Those who choose either acupuncture treatment or acupuncture-like sham will be asked to provide a reason why they have made that assumption [9].

### Monitoring

#### Data and safety monitoring plan

A Data and Safety Monitoring Board (DSMB) will be formed according to the National Institutes of Health (NIH) to monitor the study progress and review safety and quality of data [44]. The committee consists of three members, including a senior acupuncturist, a pain specialist, and a biostatistician. The DSMB is independent of the proposed study and all the committee members will have to declare any conflict of interest in the study. Regular board meetings will be held to ensure that data are collected scientifically and ethically and subjects are not exposed to unnecessary risks [44].

### Data processing and analysis

#### Data management

Data will be collected by blinded assessors. All data will be double-entered and checked for consistency prior to analyses. If an immediate entry is not possible, data collected in paper form will be scanned and coded with subjects’ codes. All the data collected will be locked, and the Personal Data (Privacy) Ordinance (CAP 486) in Hong Kong will be complied with.

#### Statistical analyses

Statistical analyses will be performed using Statistical Product and Service Solutions (SPSS) 22 (or above) for Windows (SPSS, Chicago, IL, USA) or SAS. The intention-to-treat (ITT) approach will be used. Descriptive analyses will be done on baseline demographics.

To analyze the primary outcome, AUC of the NRS pain scores at rest will be calculated by plotting on the timescale using the trapezoidal method, and the comparisons between groups will be made using the Student’s *t* test. Missing NRS scores will be replaced by linear interpolation in cases if the missing scores fall between two valid scores, or by multiple imputations if the missing score is the last pain score taken on day 5.

The NRS pain scores while coughing will be analyzed in the same way as the pain scores at rest. For other secondary outcomes, cumulative morphine assumption between the two groups will be compared with Student’s *t* test or the Wilcoxon rank-sum test; time-to-event data will be analyzed by the method of survival analysis. Median time to event will be calculated using the Kaplan-Meier product limit estimator, and log-rank test will be used to determine the statistical significance of the treatment difference in the survival curves for those time-to-event variables. Analyses of secondary outcomes are not part of the power calculations of the study, and should, therefore, be considered exploratory. All tests are two-tailed, and a *p* value of less than 0.05 is considered statistically significant. The effect size will be estimated by Cohen’s *d* which is the mean difference between groups per pooled standard deviation. The mean difference and its 95% confidence interval will also be calculated using SPSS.

### Safety analyses

The population for safety analyses will include all participants enrolled in the trial. Participants will be informed the potential adverse events related to acupuncture procedure such as bruises, hematomas, infection, pain, chondritis, during the signing of inform consent. Adverse events will be coded using the *World Health Organization (WHO) Adverse Reaction Terminology Dictionary*. The research assistant will follow-up the participant’s adverse event until it has resolved, subsided, stabilized, or the event is otherwise explained. Special attention will be given to those participants who have discontinued treatment due to adverse events or who experience serious adverse events.

### Health economic evaluation

The cost-effectiveness analysis will be performed using incremental cost-effectiveness ratio of acupuncture vs sham acupuncture, i.e., (cost of acupuncture minus cost of sham acupuncture)/(effectiveness of acupuncture minus effectiveness of sham acupuncture) as a health economic outcome.

The perspective of health service provider will be adopted when evaluating the costs of implementation of acupuncture as an adjunct treatment in the postoperative pain management in public inpatient setting. Only direct medical costs for two groups will be calculated. The costs to be included are staff time costs and treatment cost for patients. To estimate the staff time cost for making referrals, the time spent on each participant by each staff member will be recorded. The average number of minutes spent will be multiplied by the relevant salary rate per minute. The length of hospital stay will be recorded to estimate the cost by the multiplication of the number of inpatient days with inpatient hospital stay cost per day according to the most updated Government Gazette.

The acupuncture treatment cost will be estimated for each treatment in terms of acupuncturist’s time spent on delivering the treatment, including traveling time, multiplied by salary rate per minute. The cost of analgesics, acupuncture needles and other consumable will be calculated. The capital cost included the electric stimulator with 10% annual maintenance fee.

The effectiveness of acupuncture and sham acupuncture groups will be measured by quality-adjusted life expectancy using the AUC method which aggregates the PBMH value, measured by either SF-6D or EQ-5D-5 L, from baseline day to the study end day.

## Discussion

The study protocol of the proposed trial will be conducted at the Queen Merry Hospital which is under the Hong Kong HA. Unlike many hospitals in the West and in China, acupuncture has become a routine in-patient service [45–47]. However, currently, acupuncture is not available in the Hong Kong HA system. The present study is designed to investigate whether it is feasible, effective and safe to use acupuncture as an adjunctive therapy for patients who suffer from postoperative pain and discomfort. If successful, the study would provide useful information for integrating acupuncture into perioperative care as part of the effort of integrating Chinese and Western medicine in Hong Kong hospitals.

This study protocol faces several challenges and limitations. Firstly, patients with different types of gynecological neoplasia requiring laparotomy will be included, which may create variability in the population. However, as wound pain is the main target of treatment, the variation of types of neoplasm would not be a great problem. To address this issue, only patients with midline incision are recruited, which would likely involve similar degrees of wound pain being experienced by each patient. In addition, randomization will be done rigorously so that the types of neoplasm in the two groups can be balanced.

Secondly, although in many of the previous trials on postoperative abdominal surgical pain, acupuncture was performed before the induction of anesthesia in the operating theater [3, 13, 31], it is infeasible to conduct preoperative acupuncture in our operating theaters which will lengthen the interval time between preoperative acupuncture being performed and the actual operation. This is because the surgical team was concerned about the underlying risks and potential liability. Therefore, in this trial, preoperative treatment will be carried out in the ward within 2 h before the operation.

Thirdly, the surgery time is uncertain and often varies due to the nature of the various underlying diseases, making it difficult to schedule an acupuncture treatment. To solve this problem, preoperative acupuncture will be arranged based on the estimated time of the operation list and the first postoperative treatment will be done upon arrival on the ward. The exact operation time and acupuncture treatment time will be recorded for further analysis.

Fourthly, it is not appropriate to regard sham acupuncture as a mere placebo or control group, although non-insertion sham acupuncture has been chosen in this study to maximize the treatment between the two groups [48]. Treatment costs incurred in the sham acupuncture arm involve sham acupuncture needles which will be assumed to cost the same as the acupuncture needles. The treatment efficacy of the sham acupuncture will not be assumed to be the same as that for the control group. In line with a previous randomized, sham-controlled trial alongside cost-effectiveness analysis of acupressure [49], the sham arm was one of the study arms for effectiveness and cost-effectiveness evaluation. Therefore, sham acupuncture will be considered as one competing intervention in the cost-effectiveness analysis.

Though there are many difficulties, the quality of the trial will be closely monitored by the Data and Safety Monitoring Board (DSMB) which is an independent board composed of a biostatistician, a senior Chinese medicine practitioner, and an anesthesiologist. It is hoped that this study will provide high-quality evidence on the efficacy and safety of acupuncture for alleviating postoperative pain as well as useful information for health economic analysis.

We hope that our findings will advance knowledge on the effectiveness of acupuncture in routine integrative medicine practice in Hong Kong hospital settings.

### Trial status

The first patient was recruited on 12 October 2016. Patient recruitment is being continued.

## Additional file

Additional file 1: Completed SPIRIT 2013 Checklist: items addressed in this clinical trial protocol and related documents. (PDF 173 kb)

## Abbreviations

AA: Auricular acupuncture
ASA: American Society of Anesthesiologists
AUC: Area under the curve
CI: Confidence interval
Co-Is: Co-investigators
CONSORT: Consolidated Standards of Reporting Trials
DSMB: Data and Safety Monitoring Board
EA: Electroacupuncture
EQ-5D-5 L: EuroQol-5 Dimension-5 Level
HA: Hospital Authority
ITT: Intention-to-treat
*n*: Number of sample
NIH: National Institutes of Health
NMDAR: *N*-methyl-d-aspartate receptor
NRS: Pain Numerical Rating Scale
PBMH: Preference-based measure of health
PCA: Patient-controlled analgesia
PI: Principal investigator
QoR-9: Quality of Recovery- 9
SF-6D: Short Form-6 dimension
SPIRIT: Standard Protocol Items: Recommendations for Interventional Trials
SPSS: Statistical Product and Service Solutions
STRICTA: Standards for Reporting Interventions in Controlled Trials of Acupuncture
